# Correction: National debt management and business sustainability in Africa’s largest economy: A focus on the private sector

**DOI:** 10.1371/journal.pone.0320870

**Published:** 2025-03-20

**Authors:** Mengya Shang, Uchechukwu E. Okorie, Yin Hang, Xiaosong Jin, Daniel E. Ufua

An additional affiliation is missing for the fifth author. Daniel E. Ufua is also affiliated with Research Fellow, University of Religions and Denominations, Qom, Iran.
